# Thyroid Hormone Resistance: A Case Report of a Novel Missense Thyroid Hormone Receptor (THR) Mutation

**DOI:** 10.7759/cureus.77480

**Published:** 2025-01-15

**Authors:** Mst Laizuman Nahar, Ling Cui

**Affiliations:** 1 Internal Medicine, Mount Sinai South Nassau, Oceanside, USA; 2 Endocrinology, Mount Sinai Hospital, Oceanside, USA

**Keywords:** hypothyroid, thyroid disorder, thyroid hormone receptor mutation, thyroid receptor, thyroid-stimulating hormone (tsh)

## Abstract

Resistance to thyroid hormone is a rare genetic condition caused by germline mutations of the thyroid hormone receptor gene. The precise incidence is unknown; sporadic or de novo mutations are found. The mutant receptor results in an impaired thyroid hormone function. Thyroid hormone receptor beta gene (THRB) mutations and alpha gene (THRA) mutations are the main sites of mutation. Clinical features vary; they can show features of hyperthyroidism, hypothyroidism, or a combination of both. Even different tissues in the same individual may have different effects. Diagnosis is confirmed by genetic testing. The treatment is based on symptoms. Here we describe a case of thyroid hormone resistance, whose case was confirmed with genetic analysis, with a mutation in the THR gene, not found on online databases.

## Introduction

Abnormal thyroid function is a common condition. Resistance to thyroid hormone is a rare genetic condition caused by germline mutations of the thyroid hormone receptor gene that show similar patterns as thyroid stimulating hormone (TSH)-producing pituitary adenoma, high TSH, and free T4 but show mixed features of hyperthyroid and hypothyroid symptoms. The precise incidence is unknown, but according to a limited neonatal survey, the occurrence is one in 40,000 live births (La Franchi SH, 2003) [1], affecting both males and females equally. Around 75% of cases are familiar; sporadic or de novo mutations are found in 19% of cases (Dumitrescu AM, 2023) [2]. The mutant receptor results in an impaired function of thyroid hormone (McDermott MT, 1993) [3]. Thyroid hormone receptor beta gene (THRB) mutations were found in 1989, and alpha gene (THRA) mutations were found in 2012. Clinical features depend on the degree and nature of thyroid receptor protein abnormalities (Refetoff S, 1993) [4]; they can show hyperthyroidism, hypothyroidism, or a combination of both. Different tissues in the same individual may have other effects. Diagnosis is based on clinical features and laboratory findings and confirmed by genetic testing. The treatment is based on symptoms. Here, we describe a case of thyroid hormone resistance, whose case was confirmed with genetic analysis, with a mutation in the THR gene, that was not found on online databases. 

## Case presentation

The patient is an 84-year-old female with multiple comorbidities, including diabetes, hyperlipidemia, and osteoporosis; she presented to an endocrinology clinic in 2022 for a follow-up for her thyroid disease. She was adopted; her son had Graves' disease, and her daughter did not have any thyroid disease. Her lab results from her primary care physician are presented in Table 1. 

**Table 1:** Patient's lab value

Thyroid component	01/18/18	02/03/18	07/26/18	11/15/18	08/21/19	02/07/19	09/02/20	09/11/20	02/01/22	Normal lab reference
Free thyroxine level	-	2.5	2.6	2.2	2.2	2.3	-	2.5	1.5	0.8-1.8ng/dl
Total T4	-	19.4	-	-	-	18.1	-	14.2	-	5.4-11.5 mmol/l
Thyroid stimulating hormone level	34.43	27.74	23.66	26.70	-	26.42	26.59	-	31.72	0.5-5mIU/L
Triiodothyronine level	-	-	147	-	-	-	-	-	-	60-180 ng/dl
Thyroid peroxidase antibody	49	-	-	-	-	-	-	-	-	<30 IU/ml)
Thyroglobulin antibody	603	-	-	-	-	-	-	-	-	<2500IU /ml

Her labs showed high TSH and a high free T4 clinically; indicating she was hypothyroid. An MRI of the brain was done at the time to rule out a TSH-producing pituitary tumor, and the pituitary gland was normal as per the patient. 

She started levothyroxine replacement in 2018; her symptoms improved, and free T4 decreased when she came for the follow-up in 2022. The patient was maintained on levothyroxine 137 mcg. 

Genetic testing of RTH gene sequencing was performed in 2022, and she was found positive for one copy of the c.1375T>C (p. Phe459Leu) variant in the THRB gene. The resultant mutation causes a change of phenylalanine for leucine at the amino acid level of 459. 

## Discussion

Thyroid function abnormalities can be diagnosed with clinical manifestations along with abnormalities in lab findings. Abnormalities in the thyroid gland or pituitary or hypothalamic dysfunction can be differentiated with lab values. Usually, patients with thyroid hormone resistance have elevated thyroid hormone levels along with non-suppressed TSH levels, like TSH-producing pituitary tumors. Patients might have been misdiagnosed and treated inappropriately. Due to various clinical manifestations, diagnosis is challenging and requires high clinical suspicion. Case reports [5] have been published that the patient is misdiagnosed and treated as a Graves‘ disease. Approximately 170 mutations in TRβ were found; other mutations [6] are likely to exist. Genetic testing in our patients found a missense mutation in the THRB gene; this variant has not been described in online databases or reported in large, multi-ethnic general populations (Genome Aggregation Database (gnomAD) https://gnomad.broadinstitute.org). We have found only one case report, to our knowledge, to have the same locus missense mutation, in a two-year-old Chinese child, who had a de novo missense mutation in the same locus where valine replaced phenylalanine, c.1375T > G (p. Phe459Val) [7]. Treatment is focused symptomatically instead of normalizing thyroid hormone. Most patients overcome resistance by increasing thyroid hormone production, thus requiring no treatment. A supra-physiological dose of thyroid hormone might be needed in patients showing features of hypothyroidism, as elevated thyroid hormone cannot overcome peripheral resistance. For patients showing symptoms of hyperthyroidism, treatment may be directed at achieving symptom relief. In a subset of thyroid hormone-resistant beta mutations, the thyroid hormone analog triiodothyroacetic acid (TRIAC) is effective in relieving thyrotoxic symptoms, though its outcome is controversial. One case [8,9] reported that after five years of effectively controlling symptoms of thyrotoxicosis, patients developed autoimmune hyperthyroidism. Dextrothyroxine [10] is also helpful, whereas TRAIC cannot be used.

## Conclusions

This case report describes a patient with thyroid hormone resistance due to a heterozygous mutation in the THRB gene. The specific mutation is c.1375T>C, resulting in the amino acid substitution p.Phe459Leu (phenylalanine replaced by leucine at position 459). This variant has not been previously reported in any online genetic databases, suggesting it may be a novel mutation or rare in the population. Despite the presence of this mutation, the patient maintains a clinically euthyroid status with levothyroxine supplementation. This finding may provide insight into the variability of thyroid hormone resistance syndromes and the role of clinical judgment in managing such cases.
